# Broadening the Substrate Specificity of Cellobiose Phosphorylase from *Clostridium thermocellum* for Improved Transformation of Cellodextrin to Starch

**DOI:** 10.3390/ijms241914452

**Published:** 2023-09-22

**Authors:** Yuanyuan Zhang, Yapeng Li, Hui Lin, Guotao Mao, Xiang Long, Xinyu Liu, Hongge Chen

**Affiliations:** College of Life Sciences, Henan Agricultural University, Zhengzhou 450046, China; 13592690539@163.com (Y.Z.); 13503827511@163.com (Y.L.); huilin@henau.edu.cn (H.L.); maoguotao@henau.edu.cn (G.M.); 18339345957@163.com (X.L.); liuxinyu@henau.edu.cn (X.L.)

**Keywords:** cellobiose phosphorylase, cellodextrin phosphorylase, substrate specificity, cellodextrin, amylose

## Abstract

Cellobiose phosphorylase (CBP) catalyzes the reversible phosphorolysis of cellobiose into α-glucose 1-phosphate and glucose. A CBP with a broadened substrate specificity would be more desirable when utilized to convert cellulose into amylose (*PNAS*, 110: 7182–7187, 2013) and to construct yeast that can phosphorolytically use cellodextrin to produce ethanol. Based on the structure differences in the catalytic loops of CBP and cellodextrin phosphorylase from *Clostridium thermocellum* (named CtCBP and CtCDP, respectively), CtCBP was mutated to change its substrate specificity. A single-site mutant S497G was identified to exhibit a 5.7-fold higher catalytic efficiency with cellotriose as a substrate in the phosphorolytic reaction compared to the wild type, without any loss of catalytic efficiency on its natural substrate, cellobiose. When the S497G variant was used in the transformation of mixed cellodextrin (cellobiose + cellotriose) to amylose, the amylose yield was significantly increased compared to that of wild-type CtCBP. A structure change in the substrate-binding pocket of the S497G variant accounted for its capacity to accept longer cellodextrins than cellobiose. Taken together, the modified CtCBP, S497G was confirmed to acquire a promising feature favorable to those application scenarios involving cellodextrin’s phosphorolysis.

## 1. Introduction

Cellobiose phosphorylase (CBP, EC 2.4.1.20) catalyzes the reversible phosphorolysis of cellobiose into α-glucose 1-phosphate (G-1-P) and glucose. CBPs identified so far are all from anaerobic bacteria, such as *Clostridium thermocellum* [1], *Cellvibrio gilvus* [2], *Fomes annosus* [3], *Clostridium stercorarium* [4], *Thermotoga neapolitana* [5], *Cellulomonas uda* [6], *Ruminococcus albus* [7], and so on. Since G-1-P produced through the phosphorolytic pathway in vivo can be converted to G-6-P by a mutase and then enter the glycolytic pathway without being activated by a kinase, CBPs confer host bacteria with more energy advantage in utilizing cellobiose compared to the hydrolytic pathway catalyzed by a β-glucosidase.

CBPs play an important role in the biorefinery of lignocellulosic biomass. CBP, along with a cellodextrin transporter, was employed to construct an engineered *Saccharomyces cerevisiae* which could directly assimilate cellobiose to produce ethanol. Moreover, it was proved that the strains assimilating cellobiose through the phosphorolytic pathway had higher biomass and ethanol yields compared to the strains assimilating cellobiose through the hydrolytic pathway [8]. Taking advantage of the high energy product G-1-P generated by CBPs, we previously established an in vitro enzymatic pathway transforming cellulose to amylose [9]. In this one-pot transformation system, the pretreated cellulose was first partially hydrolyzed to cellobiose by selected cellulases, and then cellobiose was catalyzed by CBP from *Clostridium thermocellum* (CtCBP) to form G-1-P, which was then added to maltodextrin primers via potato alpha-glucan phosphorylase (PGP) to finally produce amylose. This in vitro starch synthesis process did not require any ATP or GTP consumption, making it easy to scale up.

**Figure 1:**
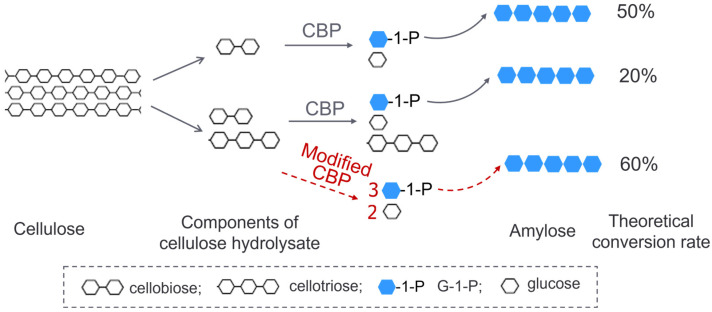
A schematic diagram showing the advantage of a modified CBP with broadened substrate specificity in the conversion of cellodextrin to starch.

Considering that CBP dominantly acts on cellobiose with little activity on cellodextrins with a degree of polymerization (DP) ≥3, the composition of cellulose hydrolysate in the above system needs to be optimized towards high content of cellobiose. Since only half of the glucose units in cellobiose were transformed into amylose, the theoretical conversion rate of cellulose to starch was only 50% given that cellulose was thoroughly degraded into cellobiose. However, a partially degraded cellulose hydrolysate obtained by chemical treatment or enzymatic treatment contains not only cellobiose, but also other cellodextrins such as cellotriose. A modified CBP with broadened substrate specificity is more desirable for the full use of cellodextrin components in the hydrolysate. Furthermore, the so-modified CBP could produce more G-1-P from cellotriose or cellotetraose than from cellobiose (two molecules of G-1-P from cellotriose and three from cellotetraose) and thus increase the conversion rate of cellulose to starch. Assuming that cellulose could be fully degraded into cellobiose and cellotriose exactly with a mole ratio of 1:1, then the theoretical conversion rate of cellulose to starch would be raised to 60% with the modified CBP, whereas the rate would be only 20% with the regular CBP (Figure 1). This theoretical calculation helps to illustrate the benefits of the modified CBP not only in the process of cellulose to starch but also in the construction of a yeast capable of producing ethanol from cellodextrin.

Based on this envisagement, here we have identified a variant of CtCBP with a broadened substrate specificity and investigated its ability to convert cellodextrin into starch.

## 2. Results

### 2.1. Modification of the Catalytic Loop of CtCBP

**Figure 2:**
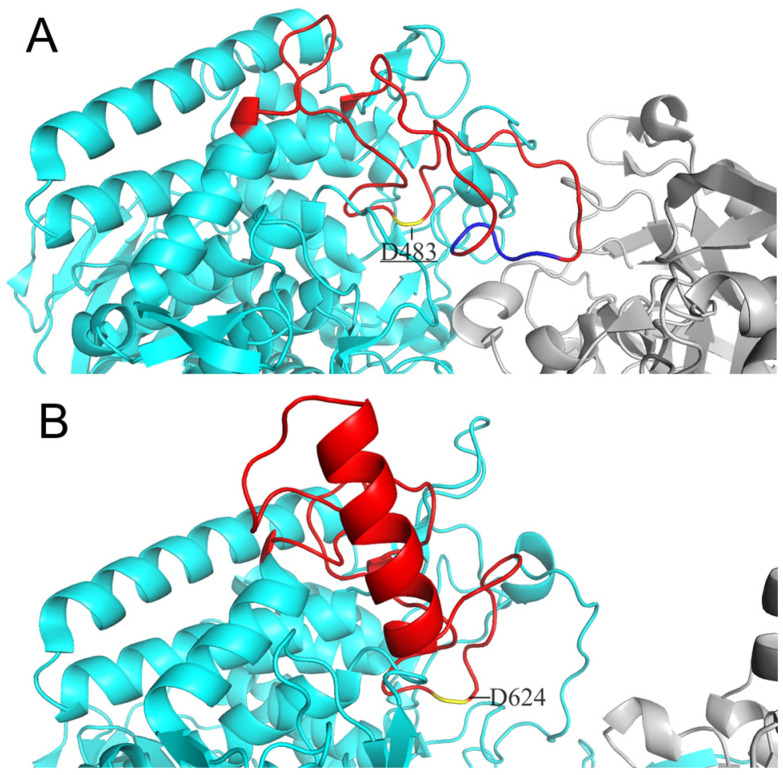
Comparison of catalytic loops between CtCBP (**A**) and CtCDP (**B**). Both catalytic loops are colored red and locations of catalytic residue D483 and D624 are marked in yellow. The portion of residues 496–499 of the catalytic loop in CtCBP is colored blue.

Considering cellodextrin phosphorylase (CDP) catalyzes the same reactions with CBP except that it acts on cellodextrins with DP ≥ 3, the structure of CDP is a good template for the substrate specificity modification of CBP. Both CDP and CBP belong to glycoside hydrolase family 94 and show high similarity in structure: both are homodimers with each subunit containing an N-terminal β-sandwich domain, a helical linker, an (α/α)_6_-barrel catalytic domain, and a C-terminal jelly roll domain [10,11]. O’Neill et al. [11] have revealed the structure features accounting for the more enclosed active site of CBP compared to that of CDP. The difference in the catalytic loop of the two enzymes was proposed to be the main factor: the catalytic loop of CBP from *Cellovibrio gilvus* (CgCBP) is fully ordered with the C-terminal portion folded over the active site pocket, while the catalytic loop of CDP from *Clostridium thermocellum* (CtCDP) forms a helix after the catalytic Asp624, going away from the active site, thereby making a relatively open dimer interface. We have also aligned the structure of CtCBP and CtCDP and found the same differences in the conformation of the catalytic loops as those found in CgCBP and CtCDP (Figure 2). We then speculate that substitution of the catalytic loop of CtCDP for that of CtCBP may broaden the dimer interface of CtCBP and hence enable CtCBP to accept longer cellodextrins as substrates.

The modification of CtCBP started with replacing the whole portion of the catalytic loop of CtCBP (residue 465–510) with the whole portion of the catalytic loop of CtCDP (residue 606–663), generating the variant designated as CBP-Δ1. Unfortunately, CBP-Δ1 lost its natural CBP activity and did not show any CDP activity, which was estimated through reversed synthetic reactions using xylose and cellobiose as glycosyl acceptors, respectively. To decrease the disturbance of catalytic loop substitution to the structure of CtCBP, we narrowed the portion of the catalytic loop needed to be replaced in CtCBP step by step and generated four other variants, as shown in Table 1. It was found that CBP-Δ5 with only four residues replaced showed the highest improved activity on cellobiose, exhibiting a two-fold specific activity compared to that of the wild type (WT). However, its natural CBP activity decreased to less than 10% of that of the wild type.

We turned to modify the key portion of the catalytic loop of CtCBP, since the catalytic loop substitution did not work as expected. It was found that residues 496–499 (ESFQ) fell into the active site pocket and the side chains might hinder the access of the larger substrates. Therefore, mutations were performed on this portion to eliminate its hindrance to the active site entrance. However, when all 4 residues were changed to glycine (named CBP-GGGG variant) or alanine (named CBP-AAAA variant), the activity on xylose was almost completely destroyed (Table 2) indicating that residues 496–499 were essential to the activity. Structural analysis showed that among the four residues, S497 and Q499 extended their side chains towards the entrance of the catalytic cavity, which might affect the access of the substrates. Due to the fact that residue Q499 formed a hydrogen bond with the catalytic residue D483, which might stabilize the catalytic residue of the enzyme (Figure S1), only residue S497 was subject to site-directed mutagenesis. The resultant variant S497G was found to display a 1.3-fold higher activity on xylose and a 2.3-fold higher activity on cellobiose compared to the wild type (Table 2), indicating that the variant obtained a broadened substrate specificity without compromising its natural activity.

### 2.2. Catalytic Properties of CtCBP Variant S497G

**Figure 3:**
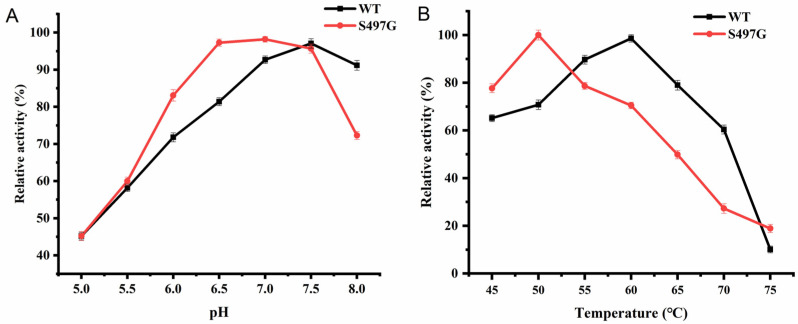
The optimum pH (**A**) and temperature (**B**) of S497G variant.

The S497G variant showed an optimal pH of 7.0, slightly lower than that of the wild type (pH 7.5), while the optimal temperature was found to be 50 °C, lower than that of the wild type (60 °C) (Figure 3). It is noteworthy that one site mutation markedly decreased the enzyme’s thermostability, although the enzyme was stable under 50 °C with 80% of initial activity remaining after 50 min of incubation. From the perspective of application, 50 °C could satisfy the majority of application scenarios that CBPs are involved in. Therefore, the decreased optimal temperature may not affect the application of S497G.

Regarding the kinetic properties of the S497G variant, the Michaelis–Menten constant (*K*_m_) and turnover number (*k*_cat_) were determined in both synthetic reactions and phosphorolytic reactions. In synthetic reactions, different concentrations of xylose or cellobiose were used as substrates. The results showed that S497G had a higher affinity as well as a higher *k*_cat_ towards both xylose and cellobiose than the wild type, indicating that it had a higher catalytic efficiency than the wild type (Table 3). In phosphorolytic reactions, when cellobiose was the substrate, S497G showed a slightly higher *k*_cat_ and catalytic efficiency compared to the wild type, while when the substrate was cellotriose, S497G had a 2.3-fold higher *k*_cat_ and a 5.7-fold higher catalytic efficiency compared to the wild type (Table 4). These changes would render variant S497G superior to the wild type under circumstances where cellodextrins of both cellobiose and cellotriose need to be phosphorolyzed.

### 2.3. Transformation of Cellodextrin to Amylose

**Figure 4:**
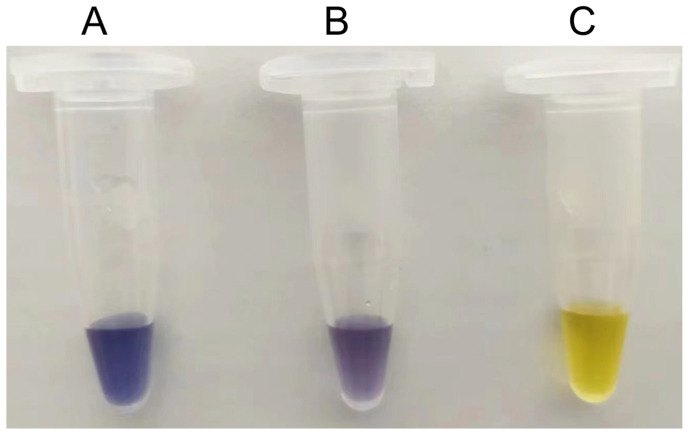
Detection of amylose formation in mixed cellodextrin system using iodine/potassium iodide solution. (**A**) S497G variant; (**B**) Wild type CtCBP; (**C**) Distilled water instead of reaction mixture.

To evaluate whether S497G could really show advantages in the transformation system of cellodextrin to amylose, a mixed cellodextrin (3% (*w*/*v*) cellobiose + 2% (*w*/*v*) cellotriose), simulating the composition of a partially degraded cellulose hydrolysate, was used as the substrate; at the same time, a transformation system with 5% (*w*/*v*) cellobiose as the substrate was used as a reference system. The enzymes S497G variant, wild-type CtCBP, and PGP were all purified to an electrophoretically homogeneous state as shown in Figure S2. All reaction conditions were kept constant between the S497G variant and wild-type CtCBP groups. We observed that in a cellobiose system, S497G variant showed a slightly increased amylose yield (15.0% vs. 13.5%) with an 11% increase in amylose yield compared to the wild type, whereas in a mixed cellodextrin system, the S497G variant produced a significantly higher amylose yield (16.2% vs. 11.7%), with a 38% increase in amylose yield compared to the wild type (Table 5). The increase in amylose yield resulting from the S497G variant compared to the wild type in the mixed cellodextrin system could also be visualized by a darker blue color when iodide solution was added (Figure 4). The difference in amylose yields between the S497G variant and the wild type is more distinct in the mixed cellodextrin system than in the cellobiose system, indicating that in the mixed cellodextrin system, S497G certainly converted more cellotriose to amylose than the wild type. The performance of S497G in the transformation system of cellodextrin to amylose confirmed that S497G possessed a broadened substrate specificity.

It is worth noting that wild-type CtCBP produced less amylose in the mixed cellodextrin system than in the cellobiose system (11.7% vs. 13.5%), which is reasonable due to a decreased concentration of CtCBP’s natural substrate, cellobiose, in the mixed cellodextrin system. In contrast, the S497G variant generated a modestly higher amylose yield in the mixed cellodextrin system than in the cellobiose system (16.2% vs. 15.0%), which demonstrated not only the capacity of the S497G variant to use cellotriose as substrate, but also the advantage of cellotriose as substrate to synthesize amylose.

### 2.4. Structure Basis for Properties of S497G Variant

**Figure 5:**
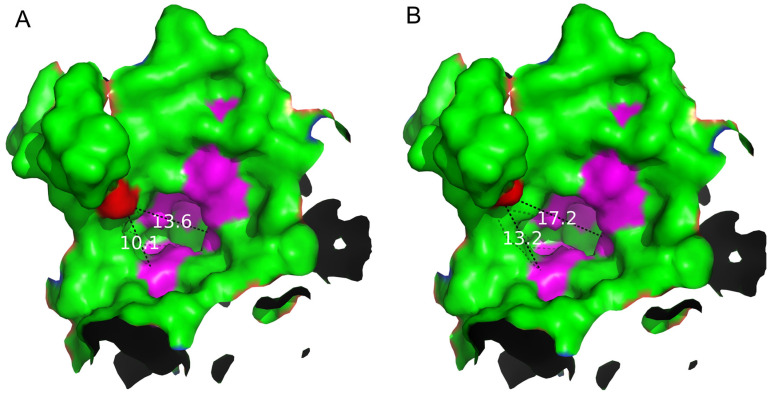
Analysis of the shape and size of the entrance of the substrate-binding pocket of the WT (**A**) and S497G variant (**B**). The mutation site S497 is marked in red. The dotted lines with values (unit: Å) represent the distances between site 497 to Gln699 and site 497 to Arg355 in the entrance of the substrate-binding pocket, respectively.

Through analyzing the structure of the S497G variant, especially the shape and the size of the entrance, we observed that the entrance of the S497G variant expanded compared to that of wild-type CtCBP due to the mutation (Figure 5). The entrance of the substrate-binding pocket of the wild-type enzyme had a diameter of 13.6 Å, while that of the S497G variant expanded to 17.2 Å, thereby enlarging the entrance size of the substrate-binding pocket, which might potentially contribute to bulky substrate entry and result in the higher catalytic activity of the S497G variant towards cellotriose.

**Figure 6:**
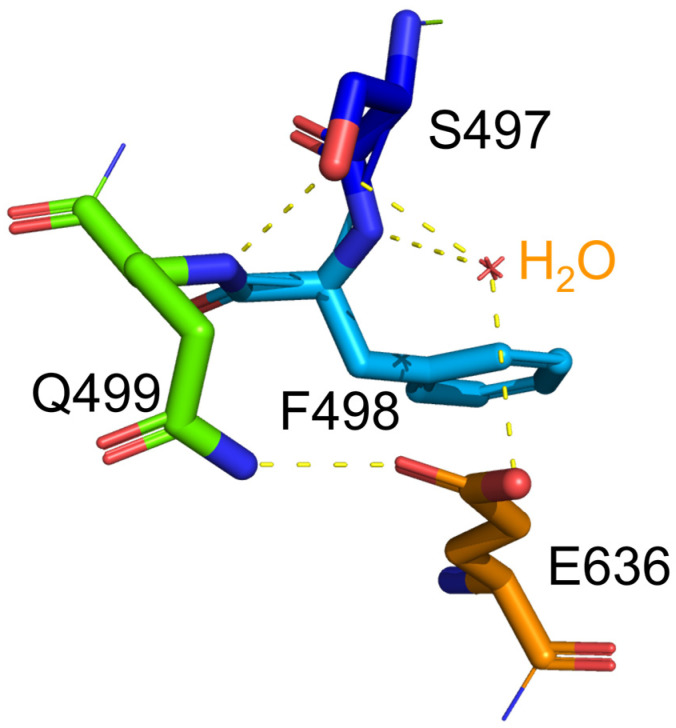
The hydrogen bonds associated with the hydroxyl group of S497 in wild-type CtCBP. Hydrogen bonds were presented as dotted lines. The hydroxyl group of S497 in WT formed one hydrogen bond with F498 and two hydrogen bonds with a water molecule.

Regarding the stability of protein, glycine substitution generally enhances the conformational flexibility of the protein [12], which corresponds to the lower stability of the protein. Indeed, the S497G variant exhibited a lower thermostability in terms of its decreased optimal temperature (50 °C). In the S497G variant, the three hydrogen bonds occurring at the hydroxyl group of serine residue in wild-type CtCBP (as shown in Figure 6) did not exist, which could make its structure not as compact as that of the wild type. It turned out that the structural change caused by S497G mutation was favorable to bulky substrate entry but not to enzyme stability.

## 3. Discussion

The phosphorolytic cleavage of cellobiose via CBP was required for the construction of *S. cerevisiae* capable of the assimilation of cellobiose as well as for the conversion of cellulose to starch. In these two applications, a CBP with the capacity to phosphorolyze cellodextrin with DP greater than 2 would be more desirable for a higher efficiency of cellulose utilization. This work focused on the modification of CBP towards broadened substrate specificity. Another strategy to solve the problem of longer cellodextrin utilization is to introduce the enzyme CDP to the system in addition to CBP. It seems reasonable despite the extra cost caused by more enzymes used. However, Ha et al. [8] demonstrated that when engineered yeasts with a cellodextrin transporter and a CDP (from *C. lentocellum* or *C. thermocellum* or *A. cellulolyticus*) were grown on YP medium containing cellobiose, cellotriose, or cellotetraose, only CDP from *C. lentocellum* showed a slight increase in cell growth compared to the control, while the other two had no effect on the host’s growth. Thus, the authors used CBP to construct the engineered yeast. Indeed, we also added CtCDP along with CtCBP when we were establishing the in vitro enzymatic pathway of cellulose to amylose, in an effort to phosphorolyze longer cellodextrins. But unexpectedly, the final oligomer formed was cellulose instead of amylose, as long as CtCDP was added to the system. The reason could be that the relatively low concentration of longer cellodextrins is not enough to drive CtCDP’s phosphorolytic reaction. On the contrary, CtCDP easily takes newly generated G-1-P as a glycosyl donor to synthesize a cellulosic oligomer which will become insoluble once DP reaches 9 [13], and the formation of this insoluble product would further drive the ongoing synthetic reaction catalyzed by CtCDP. Under these circumstances, broadening the substrate specificity of CBP would be an essential way to enhance the conversion of cellodextrin.

Regarding the modification of CBP towards the broadened substrate specificity, De Groeve et al. [14] created a variant OCP2 of *C. uda* CBP, which showed activity towards various alkyl β-glucosides, methyl α-glucoside, and cellobiose, benefiting the synthesis of cellobiosides and other glycosides. Variant OCP2 carried five mutations at sites N156/N163/T508/E649/N667, which involved residues at the substrate-binding pocket as well as residues on the route towards the entrance of the active site. Using OCP2 as a starting template, Ubiparip et al. [15] identified a new variant, OCP2_M52R, which further improved its activity towards cellobiose with a higher cellotriose yield compared to OCP2. These variants were both screened out in terms of their higher ability to use larger or anomerically substituted substrates in synthetic reactions, while the ability of these variants to use larger substrates in phosphorolytic reactions has not been evaluated. In the present work, the significant difference between the catalytic loop of CtCBP and that of CtCDP attracted our attention. Although replacing the catalytic loop of CtCBP with the corresponding fragment of CtCDP did not create the desired variants, the residue S497 at the special portion of the catalytic loop was finally identified to influence CtCBP’s substrate specificity remarkably. The variant S497G was specifically evaluated for its ability to use larger substrates in phosphorolytic reactions, and this improved ability has been validated in the conversion of mixed cellodextrin to amylose. It is notable that the residue S497 of CtCBP identified here, relevant to the substrate specificity, does not overlap with those found in *C. uda* CBP based on the alignment between the two CBPs (Figure S3).

## 4. Materials and Methods

### 4.1. Reagents

Cellobiose, maltodextrin (DE 4.0–7.0), phosphoglucomutase (PGM), glucose-6-phosphate dehydrogenase, and nicotinamide adenine dinucleotide phosphate (NADP+) were purchased from Sigma-Aldrich (St. Louis, MO, USA). Cellotriose was purchased from Megazyme Company (Bray, Ireland). Q5 High-Fidelity DNA Polymerase and Dpn1 restriction enzyme were purchased from New England BioLabs (Beijing, China) LTD.

### 4.2. Mutagenesis of CtCBP

A cellobiose phosphorylase gene from *C. thermocellum* (*ctcbp*, GenBank accession no. AAL67138.1) was originally obtained via PCR from *C. thermocellum* ATCC 27405 genomic DNA [9] and cloned to plasmid pET28a. The gene of cellodextrin phosphorylase from *C. thermocellum* (*ctcdp*) (GenBank accession no: BAA22081.1) was used as the reference sequence.

For the replacement of the CtCBP catalytic loop, first, the plasmid pET28a-*ctcbp* was linearized via inverse PCR with the removal of the fragment which needed to be replaced; second, the fragment of the CtCDP catalytic loop which needed to be introduced was synthesized and flanked by the two 20-bp homologous sequences of the 5′ end and 3′ end of the linearized plasmid; third, the above linearized plasmid and the fragment with homologous sequences were recombined using the ClonExpress^®^ II One Step Cloning Kit (Vazyme, Nanjing, China) and then transformed into *Escherichia coli*-competent cells for positive selection.

For the site-directed mutation of CtCBP, inverse PCR was used to construct a mutant plasmid. All DNA synthesis and sequencing were performed by Genewiz Inc. (Suzhou, China).

### 4.3. Expression and Purification

A potato alpha-glucan phosphorylase gene from *Solanum tuberosum* (*pgp*, GenBank accession no. D00520.1) was codon-optimized for *E. coli* and synthesized. The codon-optimized *pgp* sequence was deposited in GenBank under the accession number MZ821655. Both *ctcbp* and *pgp* were expressed in *E. coli* BL21(DE3) through plasmid pET28a with an N-terminal and a C-terminal 6×His tag. The recombinant CtCBP and PGP, as well as CtCBP variants, were purified via affinity chromatography using Ni-NTA resin as described in our previous work [16]. The protein concentration was determined using the Bradford method [17] with bovine serum albumin as the standard.

### 4.4. Enzyme Assays

In the synthetic reaction of CtCBP and its variants, the activity was assayed through measuring the amount of phosphate liberated from glycosyl donor G-1-P. For CBP activity, D-xylose was used as the glycosyl acceptor. A reaction mixture of 200 μL contained 50 μL appropriately diluted enzyme, 40 mM G-1-P, 5 mM MgCl_2_, 1 mM dithiothreitol, 10 mM D-xylose, and 50 mM Tris-HCl buffer (pH 7.5). The mixture was incubated for 20 min at 45 °C, and then the reaction was terminated through the addition of 2 mL of a molybdate reagent containing 15 mM ammonium molybdate, 100 mM zinc acetate (pH 5.0), and 500 μL of ascorbic acid reagent (10% (*w*/*v*), pH 5.0). This mixture was incubated at 30 °C for 15 min, and the absorbance was measured at 850 nm. For CDP activity, all assay protocols were the same as described previously, except that 10 mM cellobiose was used as the glycosyl acceptor and the incubation time for reaction was 6 h.

In the phosphorolytic reaction of CtCBP and its variants, the activity was assayed through measuring the formation of G-1-P from cellobiose for CBP activity or cellotriose for CDP activity [18]. A reaction mixture containing 10 mM cellobiose or cellotriose, 5 mM MgCl_2_, 1 mM dithiothreitol, 100 mM sodium phosphate buffer (pH 7.5), and appropriate enzyme was incubated for 15 min at 50 °C. The reaction was stopped via boiling for 10 min, and the amount of G-1-P produced was determined using a coupled enzyme assay measuring the formation of NADPH at 340 nm. The reaction mixture for G-1-P measurement contained phosphoglucomutase (4.0 U/mL), glucose-6-phosphate dehydrogenase (2.0 U/mL), and 3 mM NADP+ in 80 mM triethanolamine buffer (pH 7.5) containing 4.4 mM MgCl_2_. The mixture was incubated at 30 °C for 10 min, followed by spectrophotometric analysis at 340 nm.

### 4.5. Kinetic Analysis

Different concentrations of D-xylose or cellobiose (1 mM, 5 mM, 10 mM, 20 mM, 30 mM, and 40 mM) were used as substrates for determining kinetic parameters in synthetic reactions, and the initial reaction rates at each concentration of substrate were determined under the aforementioned conditions, except that the reaction was carried out at 50 °C for 10 min. For phosphorolytic reactions, different concentrations of cellobiose or cellotriose (0.5 mM, 1 mM, 2 mM, 4 mM, 5 mM, and 10 mM) were used as substrates, and the initial reaction rates at each concentration of substrate were determined under the aforementioned conditions except that the time of reaction was 10 min. *K*_m_ and *k*_cat_ values were calculated based on Hanes–Woolf plots. Each value was an average of the data from three independent determinations.

### 4.6. Transformation of Cellodextrin to Amylose

In the transformation of cellodextrin to amylose, two reaction systems were conducted: one was with 5% (*w*/*v*) cellobiose as the substrate, and the other was with 5% (*w*/*v*) mixed cellodextrin (3% (*w*/*v*) cellobiose + 2% (*w*/*v*) cellotriose) as the substrate. In both systems, 500 µL of reaction mixture contained 50 mM HEPES buffer (pH 7.5), 20 mM Na_2_HPO_4_, 75 µM maltodextrin, 30 µg/mL of CBP or S497G, and 30 µg/mL of PGP. The mixture was incubated at 45 °C for 16 h followed by 10 min of water boiling to terminate the reaction and denature enzymes. The supernatant was collected after centrifugation to determine amylose content. To qualitatively detect amylose, 100 μL of the supernatant was mixed with 10 μL of iodine/potassium iodide solution to observe the appearance of blue color. To determine the amylose yield, two volumes of 100% ethanol were added to the supernatant to precipitate amylose. Then, the precipitated amylose was separated and re-dissolved with 400 μL of deionized water. The phenol sulfuric acid method [9] was used to determine the amylose content in terms of glucose equivalent content. The amylose yield was calculated based on Equation (1) for the cellobiose system and Equation (2) for the mixed cellodextrin system. All reactions were performed in triplicate.

(1) $$Amylose\;yield\;\left(\%\right)=\frac{Amylose\;content\;\left(mg/mL\;of\;glucose\;equivalent\right)}{50\;mg/mL\times 360/342}\times 100$$

(2) $$Amylose\;yield\;\left(\%\right)=\frac{Amylose\;content\;\left(mg/mL\;of\;glucose\;equivalent\right)}{30\;mg/mL\times 360/342+20\;mg/mL\times 540/504}\times 100\;\;\;$$

### 4.7. Homology Modeling and Structure Comparison

The CtCBP structure (PDB code 3qde) and CtCDP structure (PDB code 5NZ7) were extracted from the PDB database (http://www.rcsb.org/) (accessed on 15 July 2023). The structure of the S497G variant was generated with wild-type CtCBP as a reference in PyMOL (http://www.pymol.org) (accessed on 15 July 2023). The substrate-binding pockets of CtCBP and the S497G variant were visualized using PyMOL v2.5. The size of the entrance of the substrate-binding pocket (between site 497 and Gln699, site 497 and Arg355) was measured using PyMOL.

## 5. Conclusions

A CBP with broader substrate specificity will be more desirable when used in the transformation of cellulose into amylose as well as in the construction of yeast capable of producing ethanol from cellodextrin. In this study, we engineered CBP from *C. thermocellum* and identified a variant S497G that demonstrated a 5.7-fold higher catalytic efficiency towards cellotriose in phosphorolytic reactions compared to the wild type, without any loss of catalytic efficiency on its natural substrate, cellobiose. When the S497G variant was used in the transformation of mixed cellodextrin (cellobiose + cellotriose) to amylose, the amylose yield was significantly increased, confirming its promising feature favorable to those application scenarios involving cellodextrin’s phosphorolysis. Therefore, the S497G variant can serve as a useful catalyst for the conversion of lignocellulose biomass into value-added bioproducts.

## Figures and Tables

**Table 1:** Specific activity of CtCBP variants in synthetic reaction generated by catalytic loop replacement.

Enzyme	Replaced Fragments of CtCBP	Introduced Fragments of CtCDP	Specific Activity	
			Xylose (μmol/min/mg)	Cellobiose (μmol/h/mg)
WT	/	/	2.95 ± 0.08	2.28 ± 0.04
CBP-Δ1	465–510 (46 aa)	606–663 (58 aa)	ND	ND
CBP-Δ2	484–510 (27 aa)	627–663 (37 aa)	ND	ND
CBP-Δ3	486–510 (25 aa)	627–663 (37 aa)	0.11 ± 0.01	0.58 ± 0.01
CBP-Δ4	491–510 (17 aa)	632–660 (29 aa)	0.15 ± 0.02	3.20 ± 0.11
CBP-Δ5	491–494 (4 aa)	632–650 (19 aa)	0.20 ± 0.01	4.74 ± 0.15

ND: no activity was detected.

**Table 2:** Specific activity of CtCBP variants in synthetic reaction mutated at key portion of the catalytic loop.

Enzyme	Specific Activity	
	Xylose (μmol/min/mg)	Cellobiose (μmol/h/mg)
WT	2.91 ± 0.07	2.02 ± 0.05
CBP-GGGG	0.09 ± 0.01	0.34 ± 0.02
CBP-AAAA	0.09 ± 0.01	0.16 ± 0.01
S497G	3.84 ± 0.12	4.67 ± 0.14

**Table 3:** Kinetic parameters of S497G variant in synthetic reaction.

Enzyme	Xylose			Cellobiose		
	*K*_m_ (mM)	*k*_cat_ (s^−1^)	*k*_cat_/*K*_m_ (s^−1^mM^−1^)	*K*_m_ (mM)	*k*_cat_ (s^−1^)	*k*_cat_/*K*_m_ (s^−1^mM^−1^)
WT	38.26 ± 1.40	3.25 ± 0.13	0.09	10.32 ± 1.12	0.34 ± 0.03	0.03
S497G	6.94 ± 0.38	6.39 ± 0.15	0.92	5.36 ± 0.12	0.43 ± 0.02	0.08

**Table 4:** Kinetic parameters of S497G variant in phosphorolytic reaction.

Enzyme	Cellobiose			Cellotriose		
	*K*_m_ (mM)	*k*_cat_ (s^−1^)	*k*_cat_/*K*_m_ (s^−1^mM^−1^)	*K*_m_ (mM)	*k*_cat_ (s^−1^)	*k*_cat_/*K*_m_ (s^−1^mM^−1^)
WT	8.47 ± 0.45	7.09 ± 0.46	0.84	8.65 ± 0.22	0.67 ± 0.05	0.08
S497G	6.62 ± 0.13	7.41 ± 0.35	1.12	3.48 ± 0.18	1.52 ± 0.10	0.44

**Table 5:** Starch synthesis ability of S497G variant in cellobiose system and mixed cellodextrin system.

Enzyme	Reaction System	Amylose Yield (%)
WT	Cellobiose	13.5 ± 0.3 b
S497G	Cellobiose	15.0 ± 0.2 a
WT	Mixed cellodextrin	11.7 ± 0.4 b
S497G	Mixed cellodextrin	16.2 ± 0.2 a

The different letters a and b indicate that the difference between the two enzymes in each system is significant (*p* < 0.05).

## Data Availability

The data produced in this study have been included in this manuscript.

## Supplementary Materials

The following supporting information can be downloaded at: https://www.mdpi.com/article/10.3390/ijms241914452/s1.
