# Supplementary material for: Broadening the Substrate Specificity of Cellobiose Phosphorylase from Clostridium thermocellum for Improved Transformation of Cellodextrin to Starch
Source: Int J Mol Sci. 2023 Sep 22;24(19):14452. doi: 10.3390/ijms241914452 (PMC10572201; doi:10.3390/ijms241914452)
Supplement: Supplementary file 1 [file ijms-24-14452-s001.zip › ijms-2589906-supplementary.pdf]

Supplementary Materials

Broadening the Substrate Specificity of Cellobiose Phosphorylase from *Clostridium thermocellum* for Improved Transformation of Cellodextrin to Starch

Yuanyuan Zhang, Yapeng Li, Hui Lin, Guotao Mao, Xiang Long, Xinyu Liu and Hongge Chen *

College of Life Sciences, Henan Agricultural University, Zhengzhou 450046, China
* Corresponding author: honggeyz@henau.edu.cn

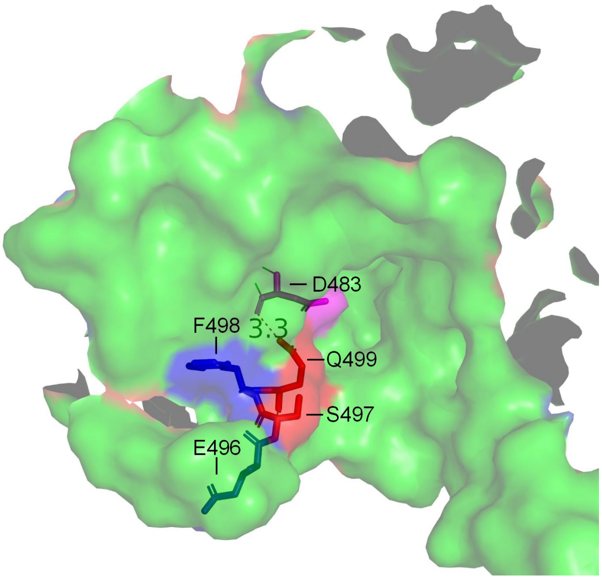

Figure S1. Structure analysis of residues 496-499. The side chains of residue S497 and Q499 are towards the entrance of the catalytic cavity, while the side chains of E496 and F498 are in the opposite direction. The value 3.3 means the distance (Å) between residue Q499 and the catalytic residue D483. The dotted line represents the hydrogen bond formed between Q499 and D483.

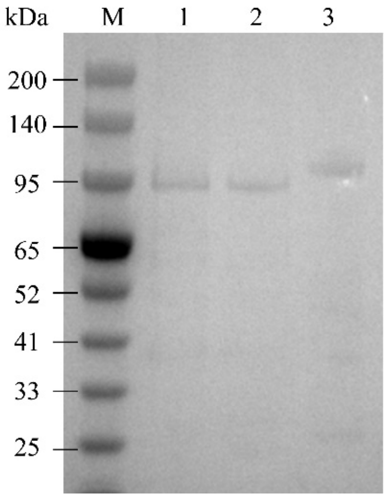

Figure S2. SDS-PAGE analysis of purified CtCBP, S497G, and PGP. Lane M—protein marker; lane 1—CtCBP; lane 2—S497G; lane 3—PGP.

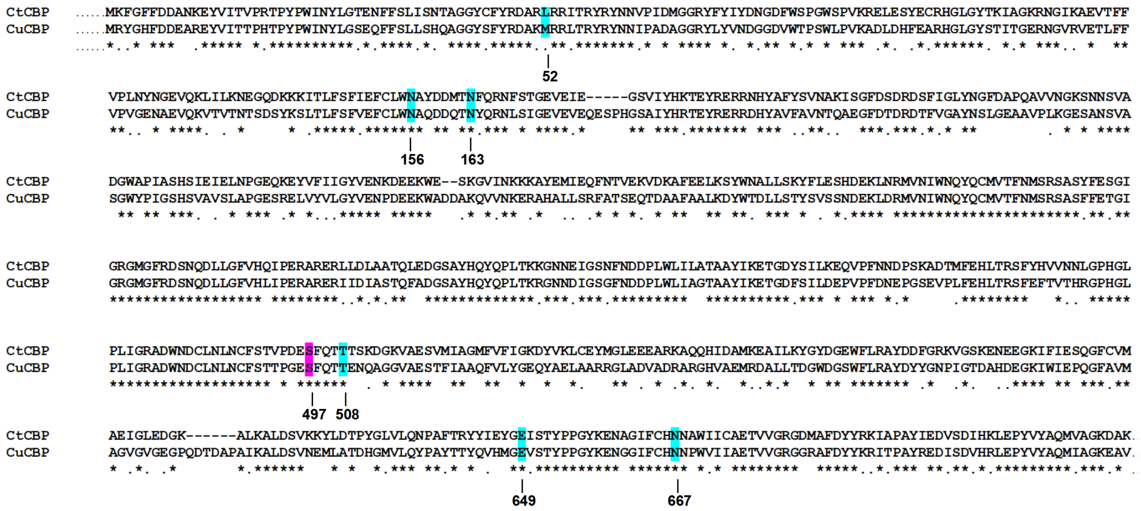

Figure S3. Sequence alignment of CtCBP and *Cellulomonas uda* CBP (CuCBP). The mutation site in CtCBP in this work is represented in purple. The mutation sites in CuCBP identified previously are represented in cyan blue.
